# Not to be loose shunted

**Published:** 2009

**Authors:** Alok Sarin

**Affiliations:** Sitaram Bhartia Institute, Delhi, India

**Figure:**
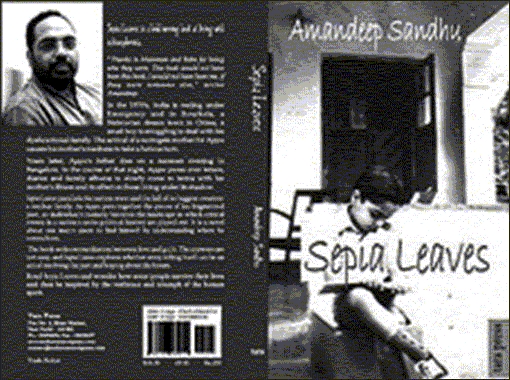


In his debut novel, *Sepia Leaves*, Amandeep Sandhu writes the following:

“Some of the coaches had ‘Not to be Loose Shunted’ written on them. I wish someone would write that on me too.

‘Not to be Loose Shunted’. I too was part of a family and should not be moved around, alone.”

In a moving, often heart-wrenching account, Sandhu describes his childhood in Rourkela and the process of growing up with a mother suffering from Schizophrenia (or *Sijofrenia,* as the child sees it). The shadow that the illness necessarily casts on childhood, parenting, family interactions, and marital relationships are seen through the wondering, often bewildered eyes of a child who does not know whether to feel angry or sad. The pragmatic, matter of fact tone of the narrative imbues it with gravity.

Describing his world with the discerning, critical eye of childhood, the author sees the main actors of his world in dispassionate, compelling detail. Bound together by marriage, but driven apart by the terrible tragedy of the illness, they struggle continuously with each other and the world, drawing the child into this in different ways. The complicated relationships within the immediate and the larger family against the fluctuating backdrop of acceptance and denial of the illness, and its behavioral sequelae, is the essence of the story. It remains as much a story about schizophrenia as it is a tale of coping with huge tragedy. The gentle demeanor of the father, his acceptance of destiny (*Sanjog*), and his resolving of issues until he is actually able to get back to enjoying his *ghazals* or an occasional drink in many ways helps the child Appu to seek his own resolution. What he first sees as weakness, emerges as strength.

Written with naïve innocence, the book does have its flaws. The title is contrived, the style often turgid, it could certainly do with more effective editing. The translations from Gurmukhi and Hindi leave much to be desired, and as in the quote from the Rani Lakshmibai poem, are sometimes inaccurate. However, the power of the narrative succeeds in carrying it through, and the journey the child makes from being overwhelmed by the illness to coming to terms with it, and more importantly, with both his parents, is the strength of the book.

In a book review for a psychiatric journal, the possible insights for the mental health professional are obviously important. What stands out here are the descriptions of symptoms, the referential thinking, the thought disorder, the behavioral changes, and more importantly, the impact of these on the fabric of family life.

The other, and to my mind more important, aspect is how peripheral the psychiatrist is to the whole process of coping with the illness. The patient, courteous doctor in the first visit, when confronted by hostility and no response to treatment soon comes across as helpless, and more worryingly, angry. He tells the family that confinement in a specialised hospital is the only answer. The family decides to take her home and care for her, and the psychiatrist appears as a distant, rigid, authoritarian figure, not as a partner in the family's endeavor to treat the illness, much less a directive influence. For many of us who routinely treat such disorders, a glimpse from the other side, may well be an eye opener. (In fact, the only psychiatrist who emerges with some credit is the shadowy and again distant figure of the Punjab-based Dr. Vig, an intriguing aside.)

The manner in which the child copes with conflicted emotions of embarrassment, shame, and the pity of neighbors and comes to a resolution is a path which many coping with psychiatric illnesses have to tread and should help others treading the same path. The fact that coping with any chronic illness means much more than just the prescription or swallowing of pills but rather the development of understanding and acceptance is something that we pay lip service to in classrooms, conference halls, and internet discussion groups. This is especially true of psychiatric illnesses with all its issues of difficult-to-understand behaviors and stigmas. The fact that more often than not the family is left to figure out the roadmap of this journey on their own is actually depressingly brought out by this narrative.

The need for everyone even remotely interested in mental health to be able to see beyond the disorder to glimpse the lives of people touched by schizophrenia should make this essential reading for all mental health professionals, and indeed, for all those interested in psychiatric illness.

